# Diagnostic possibility of the combination of exhaled nitric oxide and blood eosinophil count for eosinophilic asthma

**DOI:** 10.1186/s12890-021-01626-z

**Published:** 2021-08-09

**Authors:** Jiang-Hua Li, Rui Han, Yu-Bo Wang, Min Cheng, Heng-Yi Chen, Wen-Hui Lei, Li Li, Chen Gao, Na-Na Zhao, Nai-Fu Nie, Zhong-Yan Li, Guo-Qing Yin, Shuai Huang, Yong He

**Affiliations:** 1 Department of Respiratory Medicine, Daping Hospital, Army Medical University, 10# Changjiang Branch Road, Chongqing, 400042 China; 2 Department of Cardiology, Daping Hospital, Army Medical University, Chongqing, 400042 China; 3 Department of Clinical Laboratory Medicine, Daping Hospital, Army Medical University, Chongqing, 400042 China

**Keywords:** Asthma, Exhaled nitric oxide, Blood eosinophil count

## Abstract

**Background:**

Tests to identify reversible airflow limitation are important in asthma diagnosis, but they are time-consuming and it may be difficult for patients to cooperate. We aimed to evaluate whether the combination of fractional exhaled nitric oxide (FeNO) and blood eosinophil (B-Eos) can be used to distinguish some asthma patients who could avoid objective tests.

**Methods:**

We conducted a retrospective cohort study on 7463 suspected asthma cases between January 2014 and December 2019 in Chongqing, China, and identified 2349 patients with complete FeNO, B-Eos count, and spirometry data. Asthma was diagnosed by clinicians by the criteria of recurrent respiratory symptoms and a positive bronchial-provocation or bronchodilation test (BPT, BPD). We evaluated the diagnostic accuracy of FeNO or B-Eos alone or both in combination for asthma using receiver operating characteristic (ROC) curve analysis.

**Results:**

In this study, 824 patients were diagnosed with asthma. When FeNO and B-Eos counts were used in combination, the area under the ROC curve (AUC) for diagnosing asthma increased slightly (0.768 vs. 0.745 [FeNO] or 0.728 [B-Eos]; both *P* < 0.001). The odds ratio for having asthma increased progressively with a gradual increase in FeNO or B-Eos count (both *P* < 0.001; assessed using the Cochran–Armitage trend test). Further analysis of in-series combinations of different threshold values for these biomarkers indicated that moderately elevated biomarker levels (FeNO > 40 ppb and B-Eos > 300 cells/μl) support a diagnosis of asthma because diagnostic specificity was > 95% and the positive likelihood ratio (PLR) was > 10. This conclusion was verified when selecting the 2017–2019 data as the internal validation dataset.

**Conclusion:**

FeNO or B-Eos count alone is insufficient to accurately diagnose asthma. Patients with moderately elevated biomarkers (FeNO > 40 ppb and B-Eos > 300 cells/μl) could be diagnosed with asthma and avoid objective tests when such tests are not feasible.

**Supplementary Information:**

The online version contains supplementary material available at 10.1186/s12890-021-01626-z.

## Introduction

Asthma is characterized by recurrent respiratory symptoms and a variable expiratory-airflow limitation, affecting approximately 334 million people worldwide [1, 2]. Meanwhile, many asthma patients are still underdiagnosed, which leads to a decrease in work productivity and poor vitality and mental health [3, 4]. The main reason is that the common symptoms of asthma are relatively non-specific [5], and the objective tests recommended by the Global Initiative for Asthma (GINA), including the bronchial-provocation test (BPT) and the bronchodilation test (BDT), require complex cooperation from patients, overly long durations of examination time, and might pose certain risks [5, 6]. Therefore, finding a simple and effective method for diagnosing asthma is an urgent clinical problem.

As asthma is mainly driven by type 2 (T2: includes type 2 innate lymphoid cells [ILC2s] and T-helper 2 [Th2]) inflammatory disease [7, 8], even moderate to severe persistent corticosteroid-refractory (defined as T2-low) asthma has partial T2-high features [9]. Although induced sputum has been recommended to detect airway inflammation [1], it is time-consuming and laborious, it requires experienced laboratory personnel, and many patients cannot produce sufficient samples, making it uncommon outside specialist centers [10]. Meanwhile, fractional exhaled nitric oxide (FeNO) and blood eosinophil (B-Eos) count have been suggested as biomarkers to distinguish airway inflammation in asthma [11, 12]. Unfortunately, FeNO or B-Eos count alone is insufficient to accurately diagnose asthma [13–15]. Although previous studies showed that combining these two biomarkers provides additional predictive information [16], several limitations have been identified. In some studies, the researchers confirmed that these two biomarkers could be used to identify types of chronic respiratory diseases, but such studies were conducted in the general population [17, 18]. Significant differences in FeNO and B-Eos count between asthmatic and healthy people [19] can lead to overestimating the diagnostic accuracy of these two biomarkers. Meanwhile, some conclusions have been drawn on the basis of selected asthma patients such as adolescents and young adults [20] and might not be applicable to all adult patients. In addition, the diagnosis of asthma in some studies was self-reported (mainly based on non-specific patient symptoms) [18, 21], which led to under- or overdiagnosis of asthma because patients inadequately reported respiratory symptoms to their doctors [5]. Importantly, due to the lack of widely accepted definitions of high FeNO levels and high B-Eos counts, the combination of these two biomarkers for the diagnosis of asthma is worthy of further study.

In the present study, we diagnosed asthma when patients had recurrent respiratory symptoms and a positive result of an objective test (BPT or BDT). We studied the performance of these two biomarkers in asthmatic patients and evaluated the accuracy of FeNO or B-Eos alone or both in combination for the diagnosis of asthma. By analyzing combinations of different threshold levels of these two biomarkers, we aimed to identify which patient groups could avoid complex objective tests, which usually are not accessible in primary care.

## Methods and materials

### Population

**Fig. 1:**
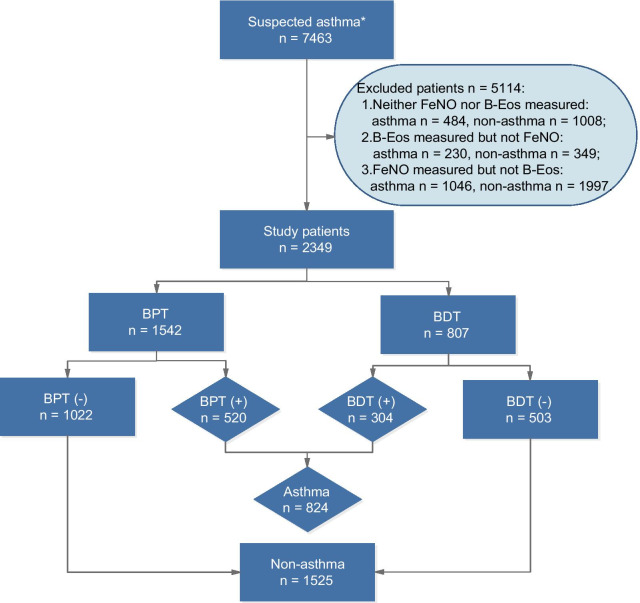
Flowchart for selecting the study population. FeNO, fractional exhaled nitric oxide; B-Eos, blood eosinophils; BPT, bronchial provocation test; BDT, bronchodilation test. *Notes*: Certain data are missing due to concerns about the extra costs of the FeNO test, pain caused by venipuncture, and the clinicians’ personal diagnosis and treatment habits. *Patients with variable respiratory symptoms such as wheezing, shortness of breath, chest tightness and cough, which vary over time and in intensity

We screened all patients who were admitted to the respiratory clinic of Daping Hospital for suspected asthma between January 2014 and December 2019. The inclusion criteria were as follows: (1) age > 12 years; (2) experienced symptoms indicative of asthma, such as wheezing, shortness of breath, chest tightness and cough, which vary over time and in intensity; (3) no respiratory infections within the past 7 days; (4) no treatment with inhaled or oral corticosteroids, leukotriene receptor antagonists, or antihistamines within the past 72 h. All examinations were prescribed simultaneously by the same clinician and completed within two days. It is important to perform the FeNO test before performing spirometry [22]; doctors performing BPT/BDT tests did not know FeNO or B-Eos results. A total of 7463 suspected asthmatic patients were screened (Fig. 1), of whom 163 patients were adolescents (under 18 years). Due to concerns about the extra costs of the FeNO test or pain caused by venipuncture to obtain blood samples to count B-Eos, as well as personal diagnosis and treatment habits of clinicians, many data were incomplete. Among them were data from subjects who did not undergo FeNO and B-Eos tests (n = 1492) and participants without FeNO (n = 579) or B-Eos measurements (n = 3043). Ultimately, 2349 patients with complete data were enrolled in the main study, and the conclusions obtained from this cohort were verified in the cohort of patients with missing or incomplete data.

### Fractional exhaled nitric oxide

FeNO was evaluated with an online measurement technique using the Nano Coulomb nitric oxide analyzer (Shangwo Biotechnology Co., Ltd., Jiangsu, China), following the recommendations from the European Respiratory Society (ERS) and the American Thoracic Society (ATS) [23]. During the inspiratory phase, the patient was required to inhale to total lung capacity through a mouthpiece, which was used to prevent air leakage and contamination by ambient nitric oxide. During the exhalation phase, an animated interface on the device helped the participant maintain a correct constant expiratory-flow rate (50 ml/s). FeNO results were reported as parts per billion (ppb), and FeNO measurements were performed prior to spirometry, the methacholine challenge test, and the reversibility test.

### Blood eosinophil count

Peripheral venous blood samples were taken, and B-Eos and leukocytes were counted using a Sysmex XN‐9000 Hematology Analyzer (Sysmex, Kobe, Japan), a multifunctional automatic hematology analyzer and leukocyte classifier. B-Eos counts were reported along with other leukocyte subpopulations, and the percentage of each subpopulation was calculated.

### Spirometry, bronchial provocation test and bronchial dilation test

Baseline spirometry, the BPT, and the BDT were performed using a Jaeger spirometer (Erich Jaeger GmbH, Würzburg, Germany) according to ATS/ERS recommendations [24]. The BPT was performed for patients whose baseline forced expiratory volume in the first second (FEV_1_) was > 70% of the predicted value, FEV_1_ was measured using a Jaeger Aerosol Provocation System [25]. Results were considered positive when the methacholine (Sigma-Aldrich, St. Louis, MO, USA) cumulative dose causing a 20% decrease in FEV_1_ (PD_20_-FEV_1_) was < 2.5 mg. The BPT was usually preferred, but the BDT was performed when the patient’s baseline FEV_1_ was < 70% of the predicted value. After the patient had inhaled 400 μg albuterol sulfate aerosol (GlaxoSmithKline, Brentford, UK), reversibility test results were considered positive when FEV_1_ was increased more than 12% and 200 ml above baseline. Positive BPT results are presented as follows: +/− indicates that the PD_20_-FEV_1_ of methacholine was 1.076–2.500 mg; + indicates that it was 0.294–1.075 mg; ++ indicates that it was 0.035–0.293 mg; +++ indicates that it was < 0.035 mg. BDT results are presented as follows: − indicates that FEV_1_ increased less than 12% or 200 ml above baseline after inhalation of 400 μg salbutamol sulfate aerosol; + indicates that FEV_1_ increased by 12–25% and its absolute value increased by 200 ml; ++ indicates that FEV_1_ increased by 25–40% and its absolute value increased by 200 ml; +++ indicates that FEV_1_ increased > 40% and its absolute value increased by 200 ml.

### Statistical analysis

Statistical analyses were mainly performed using SPSS software version 26.0 (IBM Corp., Armonk, NY, USA). Continuous variables are shown as mean ± standard deviation (SD) or as median and interquartile range (IQR), and categorical variables are presented as frequencies. Normality was evaluated using the Shapiro–Wilk test. Next, we used Student’s *t* test for normally distributed continuous variables and the Mann–Whitney *U* test for continuous non-normally distributed variables. Categorical variables were analyzed using Pearson’s chi-square test. The correlation between two non-normally distributed continuous variables was assessed by determining Spearman’s rank correlation coefficient. Receiver operating characteristic (ROC) curve analysis was performed using MedCalc software version 18.2.1 (MedCalc Software, Ostend, Belgium). The optimal cutoff values of these two biomarkers were obtained based on the highest value of the Youden index. The Hanley–McNeil non-parametric method was employed to compare the area under the ROC curve. The overlap of asthma patients with normal or elevated FeNO and B-Eos count is displayed in a Venn diagram (constructed using the online interactive Venn diagram viewer jvenn [26]). Logistic regression analysis was performed to assess risk factors for asthma or low FEV_1_. A forest plot was drawn using GraphPad Prism software version 8 (GraphPad Software, San Diego, CA, USA). The increase in odds ratio was assessed using the Cochran–Armitage trend test. *P*-values < 0.05 were considered to indicate statistical significance unless otherwise specified. To evaluate the impact of bias caused by missing data, we performed sensitivity analyses to verify whether the incomplete data population differed from the main study population, and assessed the quality of the report by using the Standards for Reporting Diagnostic accuracy studies (STARD) checklist.

## Results

### Characterization of study population

The main study population included 897 males and 1452 females, of whom 824 patients were diagnosed with asthma. As shown in Table 1, the baseline characteristics of the two groups (asthma vs. non-asthma) were identical (*P* > 0.05), while asthmatic patients had significantly higher white blood cell counts, B-Eos counts, B-Eos percentages, and FeNO levels (7.24 vs. 7.05 × 10^9^/l, 306 vs. 105 cells/μl, 4.5% vs. 1.8%, and 52 vs. 25 ppb, respectively; all *P* < 0.001). Conversely, the percentage of blood neutrophils, forced vital capacity (FVC), forced expiratory volume in the first second (FEV_1_), and FEV_1_/FVC ratio of asthmatic patients were significantly lower (59.3% vs. 61.9%, 94.4% vs. 96.2%, 79.65% vs. 92.40%, and 68.54% vs. 80.24%, respectively; all *P* < 0.001). Patients with incomplete data (n = 5114) were similar to the main study population with respect to demographic characteristics, proportion of asthma diagnoses, FeNO level, and B-Eos count (*P* > 0.05; Additional file 3: Table S1).

**Table 1:** Characteristics of the study participants (n = 2349)

Characteristics	Asthma (n = 824)	Non-asthma (n = 1525)	*P* value
Age (years)*	46 (36–53)	47 (35–55)	0.494
Height (cm)*	159 (153–165)	159 (153–165)	0.695
Weight (kg)*	59 (52–66)	59 (53–67)	0.983
BMI (kg/m^2^)*	23.38 (21.23–25.71)	23.5 (21.21–25.87)	0.250
WBC count (× 10^9^/l)*	7.24 (6.06–8.83)	7.05 (5.86–8.68)	< 0.001
%Neu (%)*	59.30 (52.50–65.70)	61.90 (55.20–68.90)	< 0.001
B-Eos count (cells/μl)*	306 (148–542)	125 (66–238)	< 0.001
%B-Eos (%)*	4.50 (2.10–7.40)	1.80 (1.00–3.30)	< 0.001
FeNO (ppb)*	52 (25–87)	25 (17–35)	< 0.001
FVC (predicted %)*	94.40 (83.40–104.38)	96.20 (86.60–106.90)	0.001
FEV_1_ (predicted %)*	79.65 (62.95–91.10)	92.40 (80.55–103.50)	< 0.001
FEV_1_/FVC (%)*	68.54 (60.4–77.35)	80.24 (73.06–84.89)	< 0.001
Sex^‡^			0.679
Female	514 (62.4%)	938 (61.5%)	
Male	310 (37.6%)	587 (38.5%)	
Objective test type^‡^			0.057
BPT	520 (63.1%)	1022 (67.0%)	
BDT	304 (36.9%)	503 (33.0%)	

Data are presented as median (interquartile range) or number (percentage)

BMI, body mass index; %Neu, percentage of blood neutrophils; WBC, white blood cell; B-Eos, blood eosinophil; %B-Eos, percentage of blood eosinophils; FeNO, fractional exhaled nitric oxide; FVC, forced vital capacity; FEV_1_, forced expiratory volume in 1 s

^*^Data were analyzed using the Mann–Whitney *U* test

^‡^Data were analyzed using Pearson’s chi-square test

### Correlation between biomarkers and BPT or BDT

**Fig. 2:**
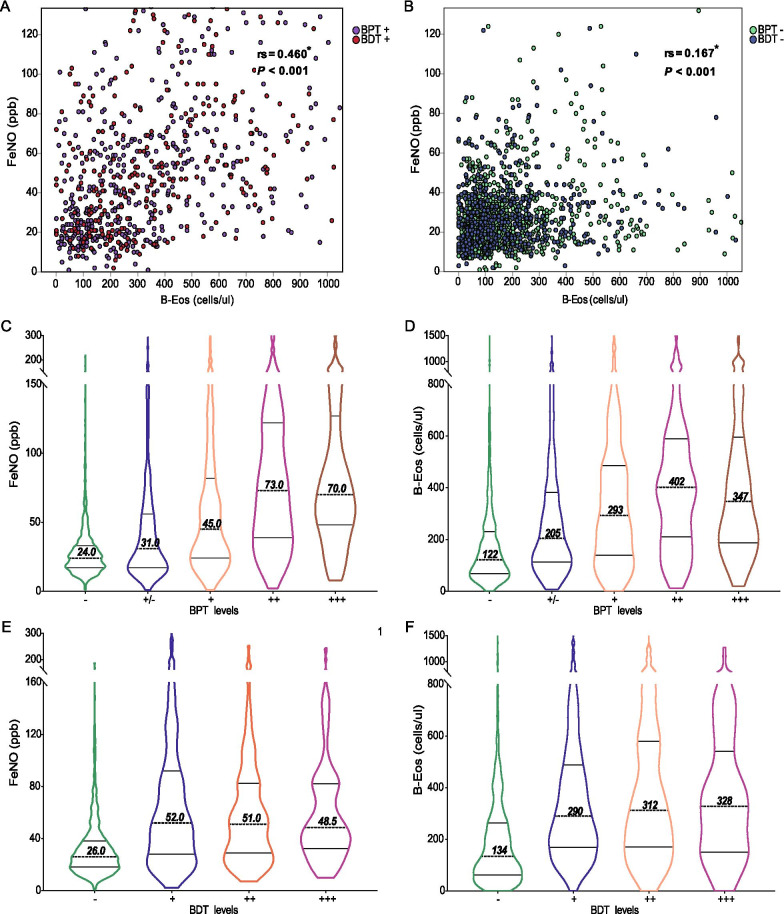
Correlation between biomarkers and objective tests. **A** Scatter plot showing the correlation between FeNO levels and the B-Eos count in asthmatic patients (n = 824). **B** Scatter plot showing the correlation between FeNO and the B-Eos count in non-asthmatic patients (n = 1525). **C** Violin plots showing the FeNO levels in different BPT subgroups. **D** Violin plots showing the B-Eos counts in different BPT subgroups. **E** Violin plots showing the FeNO levels in different BDT subgroups. **F** Violin plots showing the B-Eos counts in different BDT subgroups. rs, Spearman’s *ρ*. *Notes*: The positive BPT results are presented as follows: + / − indicates that the PD_20_-FEV_1_ of methacholine was 1.076–2.500 mg; + indicates that it was 0.294–1.075 mg; ++ indicates that it was 0.035–0.293 mg; +++ indicates that it was < 0.035 mg. The BDT results are presented as follows: − indicates that FEV_1_ increased less than 12% or 200 ml above baseline after inhalation of 400 μg salbutamol sulfate aerosol; + indicates that FEV_1_ increased by 12–25% and its absolute value increased by 200 ml; ++ indicates that FEV_1_ increased by 25–40% and its absolute value increased by 200 ml; +++ indicates that FEV_1_ increased > 40% and its absolute value increased by 200 ml. *Data were analyzed by Spearman’s rank correlation coefficients

We found a weak correlation between FeNO levels and B-Eos counts (Spearman’s *ρ* was 0.460 in asthmatic patients and 0.167 in non-asthmatic patients; both* P* < 0.001; Fig. 2A, B). When classified by airway hyperresponsiveness level (according to the cumulative dose of methacholine), FeNO and B-Eos count in the moderate to severe group were higher than in the mild group (73 or 70 vs. 45 ppb, and 402 or 347 vs. 293 cells/μl, respectively; both *P* < 0.05; Fig. 2C, D). FeNO and B-Eos count were not significantly different in any BDT-positive subgroup (grouped by the increase in FEV_1_ after the BDT; 52 vs. 51 vs. 48.5 ppb, and 290 vs. 312 vs. 328 cells/μl, respectively; all *P* > 0.05; Fig. 2E, F).

### Diagnostic capabilities of biomarkers

**Fig. 3:**
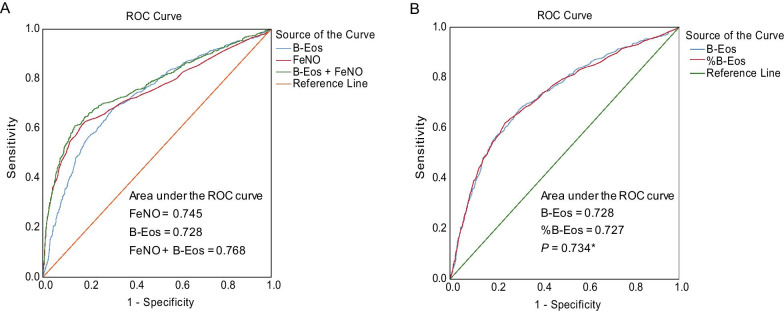
ROC curve of FeNO and blood eosinophil count for asthma diagnosis. **A** ROC curves of two biomarkers were analyzed separately or combined, a logistic regression model was used for the combination of these two biomarkers. **B** Comparison of ROC curves between the B-Eos count and the B-Eos percentage. *Data were analyzed using the Hanley–McNeil non-parametric method

The area under the receiver operating characteristic curve (AUC) of asthma diagnosis showed no difference between FeNO and B-Eos count (*P* = 0.212), but the AUC improved when these two biomarkers were used in combination (0.768 (95% confidence interval [CI], 0.746–0.789) vs. 0.745 (0.723–0.768) or 0.728 (0.706–0.750); both *P* < 0.001; Fig. 3A). The AUC of asthma diagnosis presented no significant difference between B-Eos count and percentage (0.728 (0.706–0.750) vs. 0.727 (0.705–0.749); *P* = 0.734; Fig. 3B). Whether we included incomplete data or stratified by BPT, BDT, sex (females in China hardly smoke; according to a survey on the burden of chronic diseases related to smoking, the standard smoking rate for Chinese women is 2.7% [27]), or body mass index (BMI), the diagnostic accuracy of these two biomarkers did not considerably change (Additional file 1: Figure S1A–F). According to the maximum Youden index, the optimal cutoff values for FeNO and B-Eos count to diagnose asthma were 38 ppb and 203 cells/μl, respectively; the sensitivities and specificities were respectively 62.74% and 81.44%, and 67.23% and 69.9% (Additional file 3: Table S2).

### Diagnostic accuracy for asthma of FeNO and B-Eos combined

**Fig. 4:**
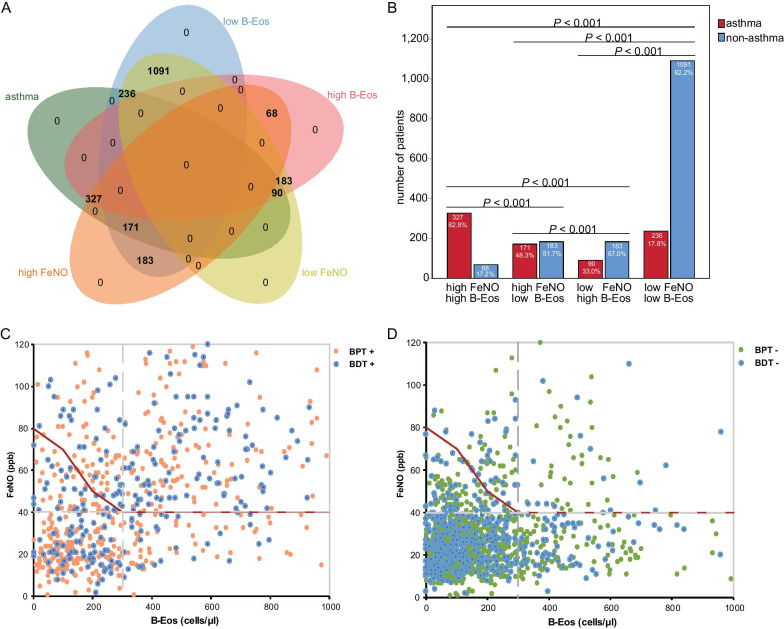
The distribution of asthma patients between high or low FeNO and B-Eos counts. **A** Venn diagram showing low or high FeNO and B-Eos counts in asthma patients. **B** Histogram analysis of low or high FeNO and B-Eos counts in asthma diagnosis. **C** Scatter plot of FeNO and B-Eos counts in asthma patients. **D** Scatter plot of FeNO and B-Eos counts in non-asthmatic patients. *Notes*: High or low FeNO were defined on the basis of a cutoff value of 40 ppb. Similarly, high or low B-Eos counts were defined on the basis of a cutoff value of 300 cells/μl. The red lines in **C**, **D** were drawn by linking different thresholds of FeNO and B-Eos counts to achieve the diagnosis goals of a positive likelihood ratio exceeding 10 and a diagnostic specificity exceeding 95%

As FeNO or B-Eos count alone was insufficient to accurately diagnose asthma, we analyzed the diagnostic accuracies of in-series combinations of different threshold levels of FeNO and B-Eos for asthma (Table 2). When linking the different thresholds of these two biomarkers to reach the goal of positive likelihood ratio (PLR) > 10 (Fig. 4C, D), which permitted a diagnosis of asthma [28], the appropriate threshold values were FeNO > 40 ppb and B-Eos > 300 cells/μl. Correspondingly, 327 patients were diagnosed, accounting for 39.7% of total asthma patients, and the misdiagnosis rate was as low as 4.5% (Additional file 3: Table S3). This conclusion was verified when selecting the data from 2017 to 2019 as the verification cohort (Additional file 3: Table S4). If we simply increased the cutoff for FeNO alone to achieve the goal (PLR > 10), the appropriate threshold value was raised to 80 ppb, and diagnostic sensitivity was reduced to 28.76% (Additional file 3: Table S2). However, using B-Eos alone could not achieve the goal of PLR > 10.

**Table 2:** Diagnostic accuracy of different combinations of threshold values of these two biomarkers (n = 2349)

Categories	Sensitivity (%)	Specificity (%)	PLR	NLR	PPV (%)	NPV (%)
FeNO > 20 ppb^†^						
B-Eos > 100 cells/μl*	69.71	64.25	1.9	0.5	51.31	79.70
B-Eos > 200 cells/μl*	56.07	81.10	3.0	0.5	61.58	77.36
B-Eos > 300 cells/μl*	41.83	89.93	4.2	0.6	69.18	74.10
B-Eos > 400 cells/μl*	31.89	93.22	4.7	0.7	71.77	71.70
B-Eos > 500 cells/μl*	22.57	95.55	5.1	0.8	73.25	69.55
FeNO > 30 ppb^†^						
B-Eos > 100 cells/μl*	58.95	80.39	3.0	0.5	61.89	78.38
B-Eos > 200 cells/μl*	47.42	89.63	4.6	0.6	71.19	75.93
B-Eos > 300 cells/μl*	35.38	94.47	6.4	0.7	77.58	73.01
B-Eos > 400 cells/μl*	26.97	96.28	7.3	0.8	79.67	70.93
B-Eos > 500 cells/μl*	19.09	97.56	7.8	0.8	80.85	69.05
FeNO > 40 ppb^†^						
B-Eos > 100 cells/μl*	50.98	90.38	5.3	0.5	74.12	77.33
B-Eos > 200 cells/μl*	41.00	94.92	8.1	0.6	81.33	74.86
B-Eos > 300 cells/μl*	30.59	97.29	11.3	0.7	85.92	72.18
B-Eos > 400 cells/μl*	23.32	98.18	12.8	0.8	87.36	70.32
B-Eos > 500 cells/μl*	16.51	98.80	13.8	0.8	88.16	68.65
FeNO > 50 ppb^†^						
B-Eos > 100 cells/μl*	43.09	93.87	7.0	0.6	79.16	75.32
B-Eos > 200 cells/μl*	34.66	96.76	10.7	0.7	85.25	73.27
B-Eos > 300 cells/μl*	25.86	98.27	15.0	0.8	89.00	71.04
B-Eos > 400 cells/μl*	19.72	98.84	17.0	0.8	90.16	69.50
B-Eos > 500 cells/μl*	13.95	99.24	18.3	0.9	90.80	68.10
FeNO > 60 ppb^†^						
B-Eos > 100 cells/μl*	35.31	96.09	9.0	0.7	83.00	73.33
B-Eos > 200 cells/μl*	28.40	97.93	13.7	0.7	88.13	71.68
B-Eos > 300 cells/μl*	21.19	98.90	19.2	0.8	91.23	69.90
B-Eos > 400 cells/μl*	16.16	99.26	21.8	0.8	92.17	68.66
B-Eos > 500 cells/μl*	11.43	99.51	23.5	0.9	92.69	67.53
FeNO > 70 ppb^†^						
B-Eos > 100 cells/μl*	28.76	97.62	12.1	0.7	86.73	71.72
B-Eos > 200 cells/μl*	23.13	98.74	18.4	0.8	90.86	70.39
B-Eos > 300 cells/μl*	17.26	99.33	25.8	0.8	93.30	68.96
B-Eos > 400 cells/μl*	13.16	99.55	29.2	0.9	94.04	67.96
B-Eos > 500 cells/μl*	9.31	99.70	31.4	0.9	94.44	67.05
FeNO > 80 ppb^†^						
B-Eos > 100 cells/μl*	24.26	98.35	14.7	0.8	88.83	70.62
B-Eos > 200 cells/μl*	19.51	99.13	22.4	0.8	92.37	69.51
B-Eos > 300 cells/μl*	14.56	99.54	31.4	0.9	94.43	68.31
B-Eos > 400 cells/μl*	11.10	99.69	35.5	0.9	95.05	67.48
B-Eos > 500 cells/μl*	7.85	99.79	38.3	0.9	95.39	66.72
FeNO > 90 ppb^†^						
B-Eos > 100 cells/μl*	19.75	98.85	17.2	0.8	90.26	69.51
B-Eos > 200 cells/μl*	15.89	99.39	26.1	0.8	93.38	68.62
B-Eos > 300 cells/μl*	11.85	99.68	36.6	0.9	95.18	67.67
B-Eos > 400 cells/μl*	9.04	99.78	41.4	0.9	95.72	67.00
B-Eos > 500 cells/μl*	6.40	99.86	44.6	0.9	96.02	66.38
FeNO > 38 ppb^‡^						
B-Eos > 203 cells/μl^‡^	42.18	94.41	7.6	0.6	80.31	75.14

^†^Baseline screening value of the former biomarker

^*^Progressively increasing cutoff values of the combined biomarkers

^‡^The optimal diagnostic cutoff value for each biomarker alone

### Overlap between asthma patients and normal or elevated biomarkers

Based on cutoff values selected for these two biomarkers (FeNO, 40 ppb; B-Eos, 300 cells/μl), we classified the 2349 patients into four groups: group A (high FeNO, high B-Eos count), group B (high FeNO, low B-Eos count), group C (low FeNO, high B-Eos count), and group D (low FeNO, low B-Eos count). The overlap between asthmatic patients and increased FeNO or B-Eos count is shown in Fig. 4A. The overall chi-square test between the four groups showed statistical differences, and there were also significant differences in the proportion of asthma patients between any two of the four groups (Fig. 4B, all *P* < 0.001). Asthma patients accounted for 82.8% of group A, while in group D the proportion of asthma patients was the lowest at 17.8%.

### Increased biomarkers caused changes in the risk of having asthma or reduced lung function

**Fig. 5:**
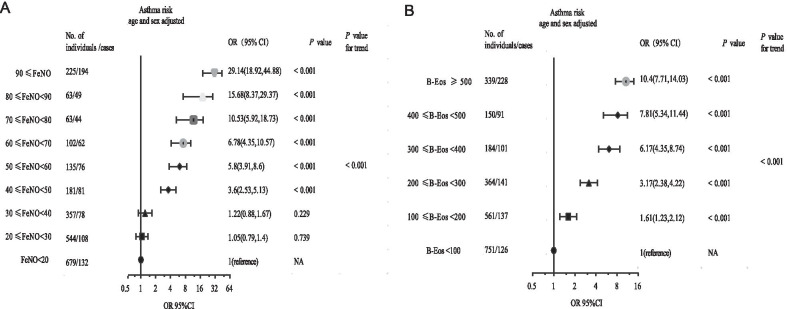
Increased biomarkers cause changes in the adjusted odds ratio for asthma. **A** Adjusted odds ratios (adjusted by age and Sex) of having asthma based on progressively increasing FeNO. **B** Adjusted odds ratios of having asthma based on progressively increasing B-Eos counts. *Notes*: Logistic regression models were used. *P* values were calculated by the Wald test. Estimates were adjusted. The Cochran–Armitage trend test was used to test whether there is a certain trend between two categorical variables. OR, odds ratio; CI, confidence interval

**Fig. 6:**
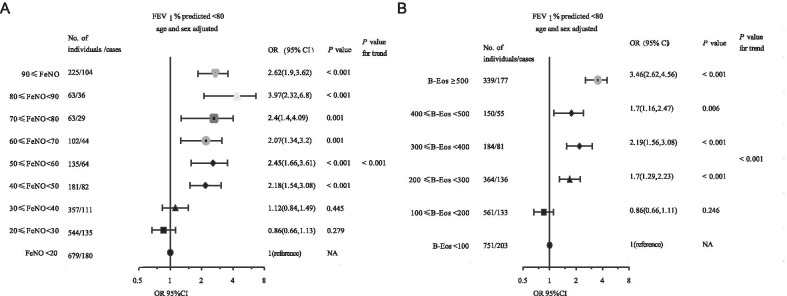
Increased biomarkers cause changes in the adjusted odds ratio of decreased FEV_1_. **A** Adjusted odds ratios of decreased FEV_1_ based on progressively increasing FeNO. **B** Adjusted odds ratios of decreased FEV_1_ based on progressively increasing B-Eos counts. *Notes* Logistic regression models were used. *P*-values were calculated by the Wald test. Estimates were adjusted. The Cochran–Armitage trend test was used to test whether there is a certain trend between two categorical variables

The multivariable adjusted odds ratio (aOR; adjusted by age and Sex) of having asthma increased progressively with a gradual increase in FeNO or B-Eos count (*P* < 0.001; Fig. 5A, B). Correspondingly, the aOR of having reduced lung function (FEV_1_ < 80% of the predicted value) increased progressively with a gradual increase of B-Eos count or FeNO (*P* < 0.001; Fig. 6A, B). These results did not significantly change when we included incomplete data (Additional file 2: Figure S2).

## Discussion

FeNO and B-Eos may help to physicians to increase the possibility of asthma diagnosis and to improve the accuracy to refer patients to a specialized center. In the present study, we provide a large-cohort analysis of the relationship among FeNO, B-Eos, and asthma in Chinese patients. According to receiver operating characteristic curve analysis, combining FeNO and B-Eos counts by logistic regression had limited effects on the diagnostic efficacy of asthma, because AUC increased from 0.745 for FeNO or 0.728 for B-Eos to only 0.768 for both biomarkers combined. Another expected finding was that the risk of having asthma increased progressively with a gradual increase in FeNO or B-Eos count. Notably, patients with moderately elevated biomarkers (FeNO > 40 ppb and B-Eos > 300 cells/μl) could be diagnosed with asthma, as the diagnostic specificity is > 95% and the PLR > 10. Although diagnostic sensitivity was reduced to 30.59%, these patients benefited from avoiding BPTs, especially since the simultaneous increase in FeNO and B-Eos count is associated with higher bronchial hyperresponsiveness [20], which may trigger a heavy bronchospasmacute [29].

At present, in order to avoid underdiagnosis or overdiagnosis, objective tests must be conducted to confirm the diagnosis of asthma [5]. However, there are several limitations to objective tests [6]. The BPT is time consuming, carries a risk of severe bronchospasm, and is generally not available in primary care; the BDT has limited value for distinguishing asthma from chronic airway diseases; and variable peak expiratory flow requires good cooperation and adherence [30–32]. Conspicuously, the UK National Institute for Health and Care Excellence (NICE) recommends that FeNO, a potential indirect measure of type 2 airway inflammation that represents the more prevalent type of asthma, should be measured in all suspected asthma patients [33]. Our data indicated that the optimal cutoff level for FeNO in the diagnosis of asthma was 38 ppb, in line with the recommendation by the Japanese Respiratory Society (JRS) [34] that a FeNO cutoff value of 35 ppb be used to diagnose asthma. Meanwhile, GINA conservatively points out that measurement of FeNO alone is insufficient to determine or rule out asthma [1]. This is because diagnostic cutoff values for FeNO are mostly concentrated in the intermediate range (25–50 ppb), and these levels can overlap extensively between asthma and other diseases [35, 36]. Elevated FeNO has previously been shown to have a rather high specificity for asthma, whereas its sensitivity is lower. The latter is probably due to the presence of non-type 2 asthma [37]. Therefore, many guidelines recommend that FeNO should be combined with other objective evidence to identify inflammatory respiratory diseases [1, 30, 33, 38].

B-Eos count, another promising and easy-to-measure biomarker, is more attractive as a means of diagnosing asthma [6, 39]. In this study, we found that the optimal diagnostic cutoff level was 203 cells/μl for B-Eos to identify asthma. Consistent with previous reports [14, 40], our data indicated that FeNO or B-Eos count alone had only moderate accuracy for diagnosing asthma, so using a single biomarker for this purpose will yield many false negatives and false positives. In all suspected asthma cases, as B-Eos count gradually increased, the risk of having FEV_1_ < 80% of the predicted value significantly increased. In addition, previous studies have demonstrated that high B-Eos counts are related to poor asthma control, risk of exacerbations, and benefits from maintenance of inhaled corticosteroids [41–43]. It should be realized that measurement of blood eosinophils can yield better risk stratification and more predictive and prognostic information in asthma [10].

Although FeNO and B-Eos are indirect measures of type 2 inflammation, these two biomarkers are regulated by different inflammatory pathways [12]. Activation of the T2 inflammatory cascade leads to secretion of various cytokines, including interleukin-4 and -13 (IL-4, IL-13), which activate nitric oxide synthase to increase FeNO in bronchial epithelial cells. IL-5 acts on IL-5 receptor subunit α (IL5RA), causing eosinophilia [11]. Similar to a previous report by Malinovschi et al. [17], FeNO was weakly correlated with B-Eos count in this study, but our data also revealed that the correlation between FeNO and B-Eos count was stronger in asthma patients than in non-asthma patients. These results suggested that the combination of FeNO and B-Eos count could help diagnose asthma. Since positive and negative predictive values depend on the prevalence of the disease irrespective of the sensitivity and specificity [28, 44], when we seek threshold levels for a diagnosis of asthma using both biomarkers, the goal is to achieve an ultrahigh PLR (> 10). When linking the different thresholds of these two biomarkers, the appropriate folding point is found at a FeNO of 40 ppb and a B-Eos count of 300 cells/μl.

Notably, our entire study population included patients with suspected early asthma who underwent the BPT and those with severe symptoms who underwent the BDT, which is more representative of suspected asthma patients in adolescents and adults. The advantage of this study was the large number of asthmatic patients who were evaluated by spirometry and standardized clinical examination. However, asthma is heterogeneous, and non–type 2 asthma is well recognized [9, 12]. Considering that asthma and chronic obstructive pulmonary disease (COPD) present with multiple overlapping phenotypes [32], it is also necessary to recognize the limitations of lung function tests in diagnosing asthma. As reported by Sano et al. [31], the overall diagnostic sensitivity of BDT for adult asthma was 0.39 and that of BPT 0.86; 5% of non-asthmatic patients had positive BPTs and BDTs. In addition, this study also had some limitations. Due to the large proportion of incomplete data, we repeated the main analysis on this incomplete data and examined the risk of selection bias. The results of these secondary analyses were similar to those of our primary analysis. Since the smoking status of the study population was unknown, we calculated the diagnostic accuracy of these two biomarkers in women (who rarely smoke in China [27]), and the results did not change significantly. This indirectly indicated that smoking status had a limited effect on the diagnostic efficiency of these two biomarkers. As this was a retrospective study, although we followed a uniform inclusion procedure, there were still potential selection biases. The atopic statuses and comorbidities of patients in this study were not fully known, which might have affected the efficiency of these two biomarkers in diagnosing asthma. These issues are worthy of further evaluation in prospective studies.

## Conclusions

There was no difference in diagnostic accuracy for asthma between FeNO and B-Eos count, and the combination of these two biomarkers could slightly improve diagnostic efficacy. Patients with moderately elevated biomarkers (FeNO > 40 ppb and B-Eos > 300 cells/μl) could be diagnosed with asthma and avoid objective tests when such tests are not feasible.

## Supplementary Information

**Additional file 1: Figure S1**. ROC curves of biomarkers for asthma diagnosis in different categories. (**A**) The ROC curve of FeNO for asthma diagnosis when including patients with incomplete data. (**B**) Comparison of ROC curves between B-Eos count and B-Eos percentage when including patients with incomplete data. (**C**) Comparison of ROC curves of these two biomarkers in patients who underwent the bronchial provocation test. (**D**) Comparison of ROC curves of two biomarkers in patients who underwent the bronchial dilation test. (**E**) Comparison of ROC curves of these two biomarkers in women (who hardly smoke). (**F**) Comparison of ROC curves of these two biomarkers in non-obese patients (BMI < 25 kg/m^2^). Abbreviations: %B-Eos, percentage of blood eosinophils; BMI, body mass index. *Data were analyzed using the Hanley–McNeil non-parametric method.

**Additional file 2: Figure S2**. Adjusted odds ratios of having asthma or decreased FEV1 in different categories when including patients with incomplete data. (**A**) Adjusted odds ratios of having asthma based on progressively increasing FeNO. (**B**) Adjusted odds ratios of having asthma based on progressively increasing B-Eos counts. (**C**) Adjusted odds ratios of having decreased FEV1 based on progressively increasing FeNO. (**D**) Adjusted odds ratios of having decreased FEV1 based on progressively increasing B-Eos counts. *Notes*: Logistic regression models were used. P values were calculated by the Wald test. Estimates were adjusted. The Cochran–Armitage trend test was used to test whether there is a certain trend between two categorical variables. Abbreviations: OR, odds ratio; CI, confidence interval.

**Additional file 3: Supplementary tables**.** Table S1**. Sensitivity analysis between patients with or without biomarker data.** Table S2**. Diagnostic accuracy of different cutoff values of biomarkers (n = 2349).** Table S3**. Diagnostic accuracy of simultaneously elevated biomarkers.** Table S4**. Diagnostic accuracy of the combination of FeNO and B-Eos count for asthma in randomly selected verification cohorts.

## Data Availability

The datasets used and/or analyzed during the current study are available from the corresponding author upon reasonable request.

## Abbreviations

FeNO: Fractional exhaled nitric oxide
B-Eos: Blood eosinophil
BPT: Bronchial-provocation test
BDT: Bronchodilation test
GINA: Global Initiative for Asthma
AUC: Area under the receiver operating characteristic curve
PLR: Positive likelihood ratio
