# Supplementary figures and images for: Diagnostic possibility of the combination of exhaled nitric oxide and blood eosinophil count for eosinophilic asthma
Source: BMC Pulm Med. 2021 Aug 9;21:259. doi: 10.1186/s12890-021-01626-z (PMC8351446; doi:10.1186/s12890-021-01626-z)

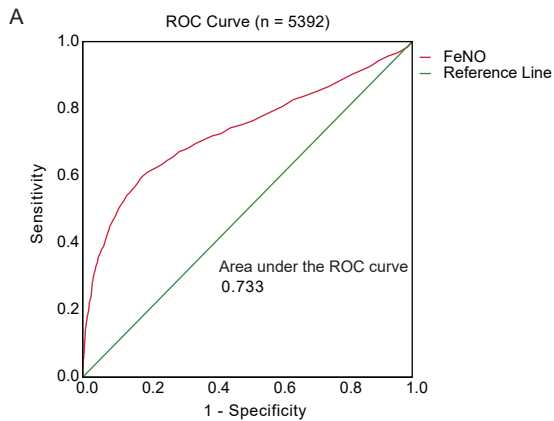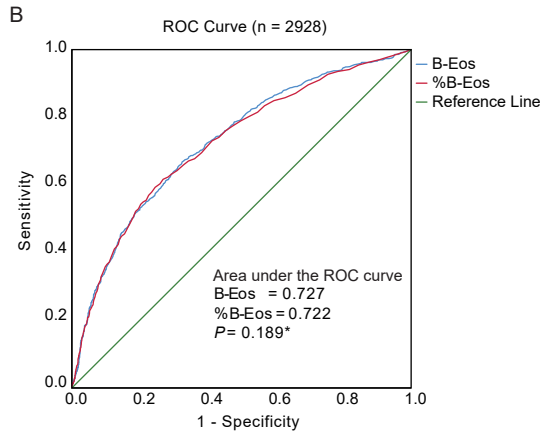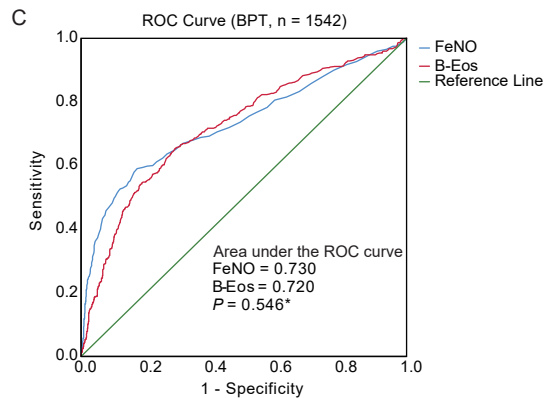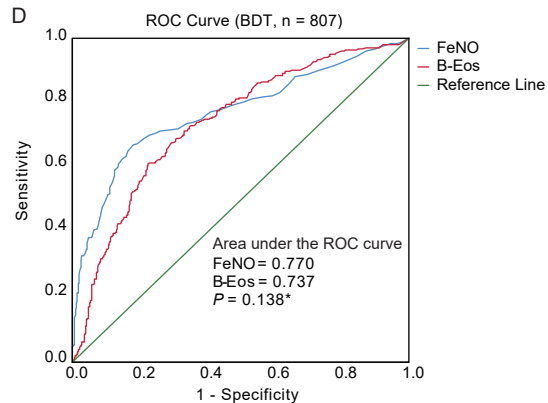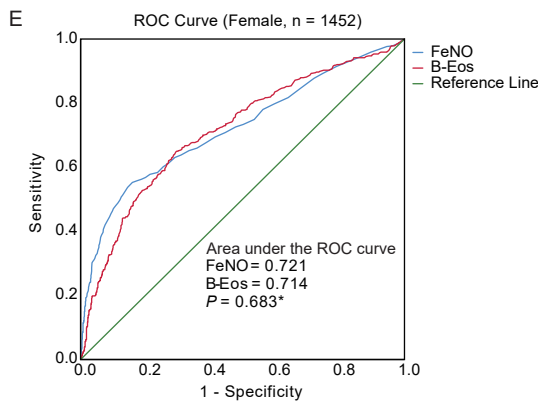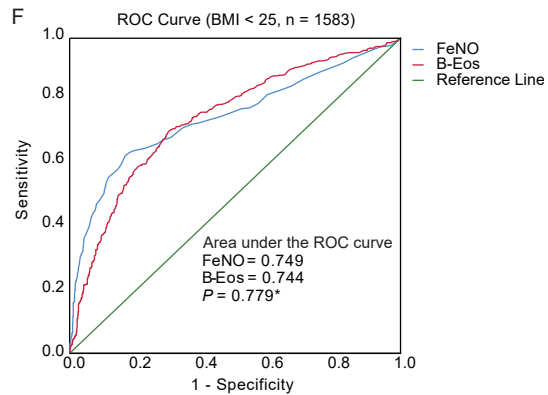

Supplement: Supplementary file 1 — Additional file 1: Figure S1. ROC curves of biomarkers for asthma diagnosis in different categories. (A) The ROC curve of FeNO for asthma diagnosis when including patients with incomplete data. (B) Comparison of ROC curves between B-Eos count and B-Eos percentage when including patients with incomplete data. (C) Comparison of ROC curves of these two biomarkers in patients who underwent the bronchial provocation test. (D) Comparison of ROC curves of two biomarkers in patients who underwent the bronchial dilation test. (E) Comparison of ROC curves of these two biomarkers in women (who hardly smoke). (F) Comparison of ROC curves of these two biomarkers in non-obese patients (BMI < 25 kg/m2). Abbreviations: %B-Eos, percentage of blood eosinophils; BMI, body mass index. *Data were analyzed using the Hanley–McNeil non-parametric method. [file 12890_2021_1626_MOESM1_ESM.pdf]

A

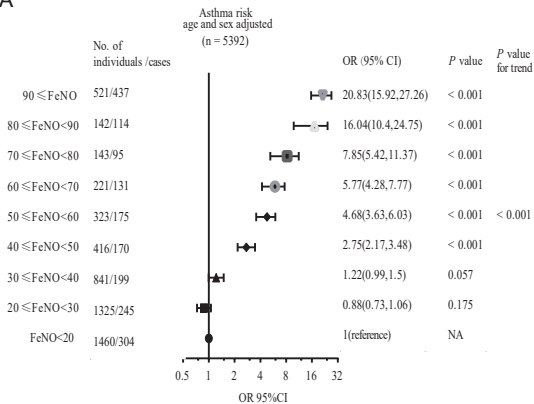

B

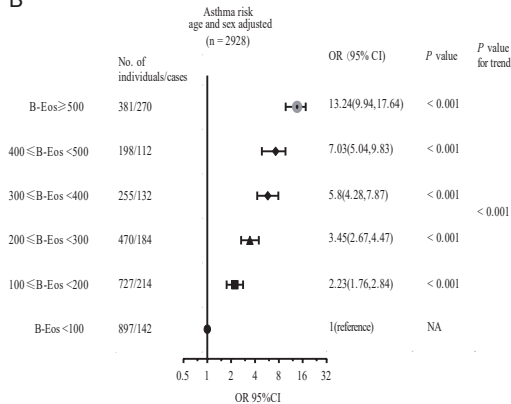

C

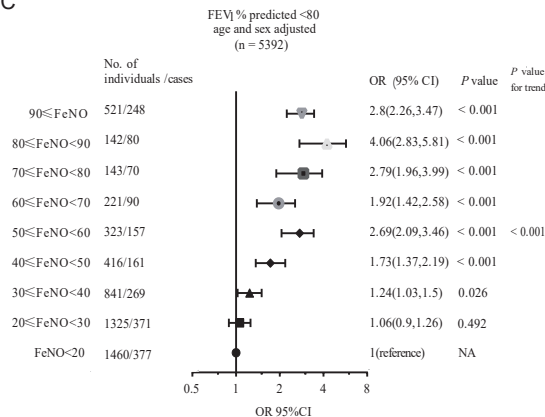

D

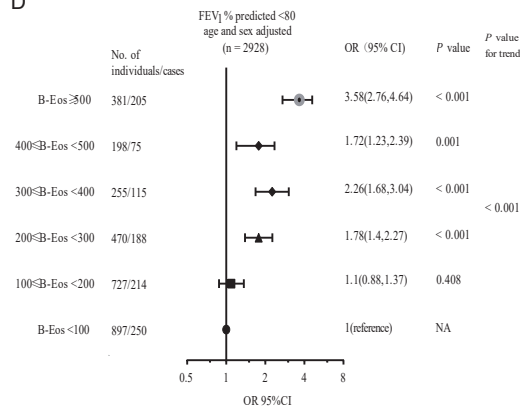

Supplement: Supplementary file 2 — Additional file 2: Figure S2. Adjusted odds ratios of having asthma or decreased FEV1 in different categories when including patients with incomplete data. (A) Adjusted odds ratios of having asthma based on progressively increasing FeNO. (B) Adjusted odds ratios of having asthma based on progressively increasing B-Eos counts. (C) Adjusted odds ratios of having decreased FEV1 based on progressively increasing FeNO. (D) Adjusted odds ratios of having decreased FEV1 based on progressively increasing B-Eos counts. Notes: Logistic regression models were used. P values were calculated by the Wald test. Estimates were adjusted. The Cochran–Armitage trend test was used to test whether there is a certain trend between two categorical variables. Abbreviations: OR, odds ratio; CI, confidence interval. [file 12890_2021_1626_MOESM2_ESM.pdf]
