# Supplementary material for: Diagnostic possibility of the combination of exhaled nitric oxide and blood eosinophil count for eosinophilic asthma
Source: BMC Pulm Med. 2021 Aug 9;21:259. doi: 10.1186/s12890-021-01626-z (PMC8351446; doi:10.1186/s12890-021-01626-z)
Supplement: Supplementary file 3 — Additional file 3: Supplementary tables. Table S1. Sensitivity analysis between patients with or without biomarker data. Table S2. Diagnostic accuracy of different cutoff values of biomarkers (n = 2349). Table S3. Diagnostic accuracy of simultaneously elevated biomarkers. Table S4. Diagnostic accuracy of the combination of FeNO and B-Eos count for asthma in randomly selected verification cohorts. [file 12890_2021_1626_MOESM3_ESM.docx]

**Supplementary appendix**

**Table S1** Sensitivity analysis between patients with or without biomarker data.

| Characteristics | Patients without FeNO­­­ or B-Eos data (n = 5114) | Patients with FeNO and B-Eos data (n = 2349) | *P* value |
| --- | --- | --- | --- |
| Age (years)* | 47 (35–55) | 46 (35–54) | 0.312 |
| Height (cm)* | 159 (153–165) | 159 (153–165) | 0.649 |
| Weight (kg)* | 59 (52–66) | 59 (53–67) | 0.858 |
| BMI (kg/m^2^)* | 23.44 (21.1–25.81) | 23.43 (21.22–25.81) | 0.991 |
| FVC (predicted %)* | 94.3 (83.9–105.34) | 95.4 (85.25–106) | 0.004 |
| FEV_1_ (predicted %)* | 86.8 (68.4–98.9) | 88 (72.6–100.6) | 0.001 |
| FEV_1_/FVC (%)* | 76.64 (64.35–83.2) | 77.4 (66.67–83.4) | 0.009 |
| WBC count (×10^9^/L)* | 7.33 (6.07–8.93) (n = 579) | 7.13 (5.93–8.75) | 0.062 |
| %Neu (%)* | 62.2 (56.2–68.9) (n = 579) | 61.1 (54.2–67.8) | 0.006 |
| B-Eos count (cells/μl)* | 182 (99–319) (n = 579) | 170 (81–350) | 0.471 |
| %B-Eos (%)* | 2.6 (1.3–4.4) (n = 579) | 2.4 (1.2–5) | 0.976 |
| FeNO (ppb)* | 29 (19–50) (n = 3043) | 29 (18–50) | 0.255 |
| Sex^‡^ |  |  | 0.056 |
| Female | 3042 (59.5%) | 1452 (61.8%) |  |
| Male | 2072 (40.5%) | 897 (38.2%) |  |
| Diagnosis^‡^ |  |  | 0.576 |
| Asthma | 1760 (34.4%) | 824 (35.1%) |  |
| Non-asthma | 3354 (65.6%) | 1525 (64.9%) |  |

**Notes:** Data are presented as median (interquartile range) or number (percentage).

*Data were analyzed using the Mann–Whitney *U* test.

‡Data were analyzed using Pearson’s chi-square test.

**Abbreviations:** BMI, body mass index; %Neu, percentage of blood neutrophils; WBC, white blood cell; B-Eos, blood eosinophil; %B-Eos, percentage of blood eosinophils; FeNO, fractional exhaled nitric oxide; FVC, forced vital capacity; FEV_1_, forced expiratory volume in 1 second.

**Table S2** Diagnostic accuracy of different cutoff values of biomarkers (n = 2349).

| Category | Sensitivity (%) | Specificity (%) | PLR | NLR | PPV (%) | NPV (%) |
| --- | --- | --- | --- | --- | --- | --- |
| FeNO (ppb) |  |  |  |  |  |  |
| > 25^†^ | 75.00 | 52.85 | 1.59 | 0.47 | 46.20 | 79.60 |
| > 38* | 62.74 | 81.44 | 3.38 | 0.46 | 64.60 | 80.20 |
| > 50^†^ | 51.09 | 89.51 | 4.87 | 0.55 | 72.50 | 77.20 |
| > 60 | 41.87 | 93.31 | 6.26 | 0.62 | 77.20 | 74.80 |
| > 70 | 34.10 | 95.93 | 8.39 | 0.69 | 81.90 | 72.90 |
| > 80 | 28.76 | 97.18 | 10.20 | 0.73 | 84.60 | 71.60 |
| > 90 | 23.42 | 98.03 | 11.91 | 0.78 | 86.50 | 70.30 |
| B-Eos count (cells/μl) | |  |  |  |  |  |
| > 203* | 67.23 | 69.90 | 2.23 | 0.47 | 54.70 | 79.80 |
| > 300^†^ | 50.61 | 83.54 | 3.07 | 0.59 | 62.40 | 75.80 |
| > 400^†^ | 38.59 | 88.92 | 3.48 | 0.69 | 65.30 | 72.80 |
| > 500^†^ | 27.31 | 92.72 | 3.75 | 0.78 | 67.00 | 70.20 |

**Abbreviations:** PLR, positive likelihood ratio; NLR, negative likelihood ratio; PPV, positive predictive value; NPV, negative predictive value.
*Optimal diagnostic cutoff value for each biomarker.

†Previously reported diagnostic cutoff value.

**Table S3** Diagnostic accuracy of simultaneously elevated biomarkers.

| Categories | Classification by BPT or BDT results | | Total |
| --- | --- | --- | --- |
|  | Asthma | Non-asthma |  |
| FeNO > 40 ppb and B-Eos > 300 cells/μl | 327 (39.7%) | 68 (4.5%) | 395 (16.8%) |
| FeNO ≤ 40 ppb or B-Eos ≤ 300 cells/μl | 497 (60.3%) | 1457 (95.5%) | 1954 (83.2%) |
| Total | 824 (100%) | 1525 (100%) | 2349 (100%) |

Patients suspected to have asthma were those with respiratory symptoms such as wheezing, shortness of breath, chest tightness, and cough, which vary over time and in intensity. The diagnosis of asthma was based on variable respiratory symptoms and positive BPT or BDT results.

**Table S4** Diagnostic accuracy of the combination of FeNO and B-Eos count for asthma in randomly selected internal verification datasets.

| Categories | Classification by BPT or BDT results | | Total |
| --- | --- | --- | --- |
|  | asthma | non-asthma |  |
| 2017 |  |  |  |
| FeNO > 40 ppb and  B-Eos > 300 cells/ul | 77 (38.12%) | 23 (5.09%) | 100 (15.29%) |
| FeNO ≤ 40 ppb or  B-Eos ≤ 300 cells/ul | 125 (61.88%) | 429 (94.91%) | 554 (84.71%) |
| Total | 202 (100%) | 452 (100%) | 654 (100%) |
| 2018 |  |  |  |
| FeNO > 40 ppb and  B-Eos > 300 cells/ul | 79 (36.92%) | 15 (3.61%) | 94 (14.94%) |
| FeNO ≤ 40 ppb or  B-Eos ≤ 300 cells/ul | 135 (63.08%) | 400 (96.39%) | 535 (85.06%) |
| Total | 214 (100%) | 415 (100%) | 629 (100%) |
| 2019 |  |  |  |
| FeNO > 40 ppb and  B-Eos > 300 cells/ul | 53 (37.06%) | 8 (5.41%) | 61 (20.96%) |
| FeNO ≤ 40 ppb or  B-Eos ≤ 300 cells/ul | 90 (62.94%) | 140 (94.59%) | 230 (79.04%) |
| Total | 143 (100%) | 148 (100%) | 291 (100%) |
